# Transcriptomic time course of skeletal muscle disuse and rehabilitation in middle‐aged adults

**DOI:** 10.14814/phy2.70497

**Published:** 2025-08-07

**Authors:** Zachary D. Von Ruff, Sean P. Kilroe, Erik D. Marchant, Emily J. Arentson‐Lantz, Steven Widen, Jill Thompson, Alejandro Villasante‐Tezanos, Elena Volpi, Doug Paddon‐Jones, Blake B. Rasmussen

**Affiliations:** ^1^ Department of Nutrition & Metabolism University of Texas Medical Branch Galveston Texas USA; ^2^ Barshop Institute for Longevity & Aging Studies, University of Texas Health Science Center at San Antonio San Antonio Texas USA; ^3^ Department of Cellular & Integrative Physiology University of Texas Health Science Center at San Antonio San Antonio Texas USA; ^4^ Next Generation Sequencing Core Facility University of Texas Medical Branch Galveston Texas USA; ^5^ Department of Biostatistics & Data Science University of Texas Medical Branch Galveston Texas USA

**Keywords:** aging, disuse atrophy, resistance exercise, transcriptomics

## Abstract

Disuse drives rapid muscle atrophy and metabolic dysfunction. This study aimed to characterize phenotypic and transcriptomic skeletal muscle changes in middle‐aged individuals during disuse and rehabilitation. Eleven healthy middle‐aged adults (6 males, 5 females; age; 57 ± 5 years) underwent 7 days of unilateral lower limb suspension (ULLS). Following disuse, participants participated in a rehabilitation program consisting of either a lower‐body resistance exercise (RE) or walking control (WC) three times weekly for 2 weeks. Bilateral skeletal muscle biopsies were collected at Day 0 and Day 7 of disuse and 2 h post‐exercise on Days 7, 9, 11, and 21. Strength testing was conducted, and RNA sequencing was performed on muscle samples. Seven days of disuse reduced knee extension strength (14%; *p* < 0.05) and isometric force (13%; *p* < 0.05). Over‐representation analysis revealed a downregulation of mRNAs related to cellular respiration and NADH dehydrogenase complex assembly. Resistance exercise induced robust, but different, transcriptional changes in both disuse‐ and control‐legs. Walking had minimal effect on the muscle transcriptome. We conclude that 7 days of disuse reduced leg strength, decreased mitochondrial gene expression, and increased inflammation and apoptosis‐related genes. We also conclude that resistance exercise enhanced recovery from disuse by improving strength, associated with significant transcriptomic changes.

## 1 INTRODUCTION

Skeletal muscle disuse resulting from immobilization, bed rest, hospitalization, or prolonged inactivity is associated with a myriad of deleterious effects on musculoskeletal health, including compromised muscle mass, function, and metabolic control. These changes can occur rapidly, with significant reductions in muscle protein synthesis, muscle size, muscle strength, and insulin sensitivity occurring after just 1 week of disuse, which are exacerbated by aging (Dirks et al., 2016; English et al., 2016). These changes have clinical significance as well. Notably, over 70% of hospitalized adults are discharged below their preadmission level of function, and many experience long‐lasting physical and metabolic impairments (Covinsky et al., 2003; Hirsch et al., 1990). Early‐phase rehabilitation following skeletal muscle disuse represents a crucial window of opportunity for promoting recovery and preventing long‐term functional decline. This is especially important in older populations, as disuse can exacerbate the age‐associated loss of skeletal muscle mass and function (English & Paddon‐Jones, 2010). Previous studies have shown that older adults' ability to recover to baseline following disuse is attenuated, even with rehabilitation, and is characterized by smaller increases in skeletal muscle cross‐sectional area and lower rates of force development than their younger counterparts (Hvid et al., 2010; Suetta et al., 2009).

Previous studies of inactivity and rehabilitation have limited application due to their use of primarily young populations, limited enrollment to men, and/or adoption of a basic “*pre‐post design*” to assess outcomes (Dirks et al., 2016; Wall et al., 2016; Phillips et al., 2009; Blakemore et al., 1996). While not without merit, these approaches lack the ability to capture early preclinical, molecular, and metabolic changes that precede and drive recovery. Data from our group and others demonstrate that disuse atrophy occurs most rapidly during the initial few days of inactivity (Fearon et al., 2011; Fielding et al., 2011; MacInnis et al., 2017; Paddon‐Jones et al., 2004; Roshanravan et al., 2017; Suetta et al., 2009). Similarly, there is often a narrow window when clinical populations have access to structured or supervised rehabilitation (Blakemore et al., 1996; Middleton et al., 2016). Therefore, our goal was to characterize phenotypic and transcriptomic skeletal muscle changes in middle‐aged men and women during disuse and rehabilitation. We also sought to determine what mode of exercise (e.g., resistance exercise or walking control) would enhance muscle recovery following short‐term disuse.

## 2 MATERIALS AND METHODS

### 2.1 Participants

Eleven healthy midlife adults (6 males, 5 females; 57 ± 5 years, BMI; 29 ± 5 kg·m^−2^) were included in the present study. Prior to enrollment, participants attended a comprehensive medical screening at the University of Texas Medical Branch (UTMB) in Galveston, TX. Eligibility for participation was assessed by a licensed medical professional. Eligibility criteria included males and postmenopausal females aged 50–65 years, BMI between 18.5 and 30 kg·m^−2^, and no existing medical conditions. Women taking hormone replacement therapy were excluded. All participants provided written consent and were compensated for their time and effort. This study was conducted in accordance with the Declaration of Helsinki and approved by the UTMB's Institutional Review Board. This study was registered at clinicaltrials.gov (NCT04151901). Baseline subject characteristics are presented in Table 1.

**TABLE 1:** Baseline characteristics of middle‐aged participants that completed 7 days of unilateral limb immobilization.

Baseline characteristics	Men (*n* = 6)	Women (*n* = 5)
Age, years	59 ± 4	55 ± 5
Height, m	1.7 ± 0.1	1.6 ± 0.1
Weight, kg	90.5 ± 15.0	67.7 ± 10.1
BMI, kg/m^2^	31.0 ± 4.0	26.5 ± 4.5
Race, (*n*)	White (4)	White (4)
	Black (1)	Asian (1)
	Asian (1)	
Ethnicity, (*n*)	Non‐Hispanic (5)	Non‐Hispanic (4)
	Hispanic (1)	Hispanic (1)

*Note*: Values are means ± SD.

Abbreviation: BMI, body mass index.

### 2.2 Study design

#### 2.2.1 Run‐in phase

**FIGURE 1:**
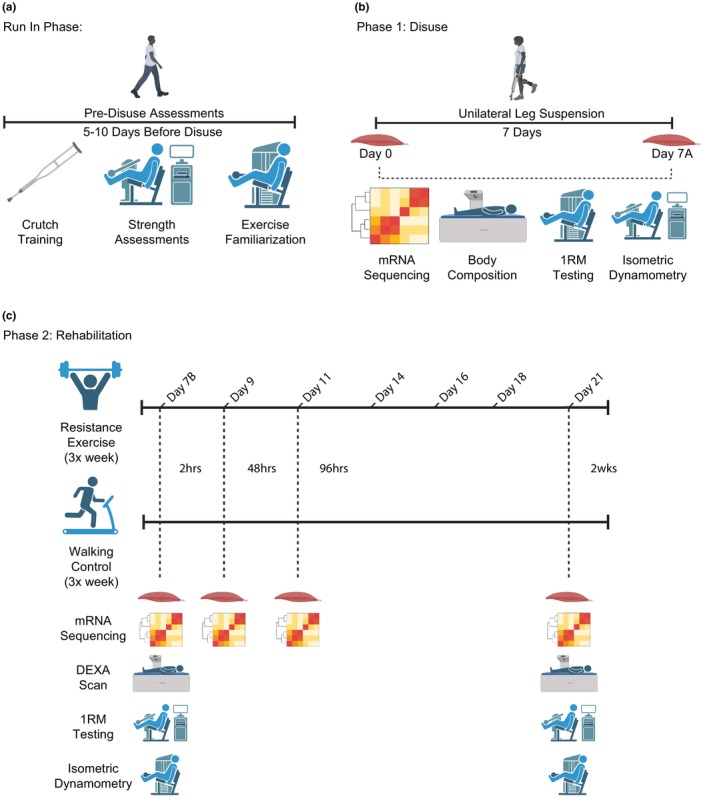
Experimental overview. (a) Human participants who were 50‐ to 65‐year‐old volunteered for a study in which body composition (via DEXA), leg strength (via 1RM and isometric dynamometry), and muscle mRNA expression were analyzed pre‐disuse, post‐disuse, and post‐rehabilitation. (b) Following the run‐in phase, participants were fitted with a leg sling and underwent 7 days of ULLS. Body composition, knee extension 1RM, and knee extension isometric strength were recorded, and skeletal muscle biopsies of the vastus lateralis were obtained on Day 0 and Day 7A. (c) Following disuse, participants were randomized to 3× per week of either resistance exercise or treadmill walking for 14 days. Body composition, knee extension 1RM, and knee extension isometric strength were collected on Day 7A and Day 21. Bilateral muscle biopsies were taken 2 h post rehabilitation on days 7B, 9, 11, and 21. 1RM, one‐repetition maximum; RE, resistance exercise; WC, walking control.

The run‐in phase included three preliminary visits to train participants in the use of the leg sling and crutches and perform baseline testing (Figure 1a). During the first two visits, participants were fitted for a leg sling and received instructions from a licensed physical therapist and an exercise professional on how to safely use walking assistive devices (i.e., crutches and walker) and proper technique for the exercises they were required to perform throughout the study. Additionally, participants were instructed to maintain their current diet and refrain from any vigorous physical activity for the remainder of the study. Approximately 5–10 days before the start of the disuse phase, participants completed a pretesting visit where baseline unilateral leg extension strength and maximal knee isometric force output were determined via one repetition maximum (1RM) and isokinetic dynamometry, respectively.

#### 2.2.2 Disuse phase

The disuse phase included 7 days of unilateral lower limb suspension (ULLS) (Figure 1b). Following an overnight fast, participants reported to UTMB the morning of the start of the disuse phase (Day 0) where baseline body composition was assessed via dual x‐ray absorptiometry (DEXA, E Lunar Prodigy Healthcare Corp, Madison, WI, USA, 2006) and bilateral skeletal muscle biopsies of the *m vastus lateralis* were obtained under local anesthetic (2% lidocaine) using the Bergstrom technique (Bergstrom, 1975) by a licensed medical professional. All skeletal muscle samples were frozen in liquid nitrogen and stored at −80°C. A blood sample was obtained via venipuncture of the antecubital vein concomitantly with the muscle biopsies. Participants were fitted with a leg sling and provided crutches or a walker to start the disuse phase. Participants were asked to return to UTMB after 4 days of ULLS where the disused leg was inspected for signs of a deep vein thrombosis (DVT) and a blood draw was obtained to check D‐dimer levels. Additionally, participants were asked to complete a ULLS adherence questionnaire. Following 7 days of ULLS, participants returned to UTMB for a DEXA scan, bilateral biopsies, and strength testing to determine disuse‐induced body composition, transcriptome, and strength changes, respectively (Day 7A).

#### 2.2.3 Rehabilitation phase

Immediately following the disuse phase, participants were randomized into either a lower‐body resistance exercise group (RE; 3 males, 3 females) or a walking control group (WC; 3 males, 2 females) and promptly began their first rehabilitation session (Day 7B) (Figure 1c). Rehabilitation sessions were performed three times per week on nonconsecutive days. To determine the acute transcriptional changes that follow rehabilitation, bilateral biopsies were obtained 2 h postexercise as previously described. Participants then returned 48 h (Day 9) and 96 h (Day 11) post‐disuse where they completed a rehabilitation session, and bilateral biopsies were obtained 2 h postexercise. Participants returned the following week and completed rehabilitation sessions on Day 14, Day 16, and Day 18. The final rehabilitation session was completed 2 weeks post‐disuse (Day 21) where the participants' body composition, knee extension 1RM, and isometric strength were reassessed. Additionally, bilateral biopsies were obtained 2 h following the last rehabilitation session.

### 2.3 Strength testing

Functional assessments of strength were determined via unilateral knee extensor one‐repetition maximum (1RM) and isometric unilateral force production. For the 1RM test, participants completed 5 min of moderate‐paced treadmill walking for a warmup. Following the warmup, participants performed several sets of unilateral knee extensions starting with a low weight for 10 repetitions. For each subsequent set, the weight was increased by ~20% and the repetitions were reduced until a 1RM was achieved. In addition, 60° knee extension isometric strength was determined via dynamometry (Biodex; Biodex Medical Systems, Shirley, New York, USA). Participants performed three repetitions at maximal effort with 90 s of rest between attempts. The average of the three repetitions was used to determine maximal isometric knee extension strength.

### 2.4 Immobilization protocol

This study utilized the ULLS model for immobilization. This method is an effective method for inducing muscle atrophy in the immobilized leg and allows the non‐immobilized leg to serve as a control group (Suetta et al., 2009). A leg sling was fitted to the participant's left leg and consisted of a waistband and an ankle cuff attached by a resistance band that held the left leg at ~30° of flexion. The left leg was selected for this study to allow participants to maintain the ability to drive an automatic transmission vehicle. Participants were instructed to always wear the leg sling except when sleeping or showering. Participants were provided with a shower chair to maintain non‐weight bearing status on the immobilized leg. Additionally, participants were provided with either crutches or a walker to assist in their usage of the non‐immobilized leg.

### 2.5 Rehabilitation protocol

For the RE group, each rehabilitation session began with a 5‐min treadmill walking warmup. Following the warmup, participants performed two unilateral lower‐body resistance exercises including leg extensions and leg curls. Both exercises were prescribed at 4 sets of 10 repetitions at 70% of 1RM, with a 2‐min rest period between sets. A warmup set of 10 repetitions at a low weight was used before the start of each exercise. Weight for subsequent sets was determined by the number of repetitions completed in the previous set. Criteria for weight selection were as follows: <8 repetitions completed; reduce weight by 5 lb., 8–12 repetitions completed; no change for the next set, >12 repetitions completed; increase weight by 5 lb. For the WC group, participants were equipped with a chest strap heart rate monitor and completed a 5‐min low‐intensity bout of treadmill walking as a warmup followed by a 30‐min moderate‐intensity bout of treadmill walking. Participants were instructed to maintain a heart rate of 100 bpm throughout the exercise session. The reason for including the WC group in this study, as opposed to a control group allowing individuals to return freely to mobilization, was to eliminate any risk that subjects would inadvertently decrease their physical function post‐intervention and fail to regain previous levels of mobility.

### 2.6 Skeletal muscle mRNA profiling

Approximately 20–30 mg of frozen muscle tissue was homogenized in TRIzol™. Following homogenization, total RNA was extracted using a guanidinium thiocyanate‐phenol‐chloroform based method (Chomczynski & Sacchi, 1987). RNA concentration and purity were determined by spectrophotometry (NanoDrop 2000; Thermo Scientific, Waltham, Massachusetts). Once extracted, RNA samples were assessed for quality by the Next Generation Sequencing and Bioinformatics Core at UTMB and then processed for library preparation and sequencing. The SMART‐3SEQ method was used for library preparation (Foley et al., 2019). Samples were sequenced on an Illumina NextSeq 550 High Output Flow Cell with the single‐end 75 base protocol following a previously described protocol (Ilinykh et al., 2022).

### 2.7 Differential mRNA expression, gene ontology, and gene set enrichment analyses

Sequence reads for each individual sample were aligned to the *H. sapiens* reference genome hg38 using the Spliced Transcript Alignment to a Reference (STAR) software version 2.7.1a (Dobin et al., 2013). FastQC was used for quality control. FeatureCounts was used to determine the number of RNA‐sequencing reads that uniquely mapped to annotated genes (Liao et al., 2014). Normalization of the reads from each sample and differential gene expression analysis was performed using the DESeq2 1.40.2 software package (Love et al., 2014), which adjusts *p* values using the Benjamini–Hochberg method, also known as False Discovery Rate or FDR. Transcripts with an FDR adjusted *p* value <0.1 and an absolute log_2_ fold change (log_2_FC) >0.4 were considered significantly differentially expressed (DE). The FDR and log_2_FC cutoffs were chosen in part because this study included a relatively small sample size (*n* = 11 subjects). As a result, these cutoffs reduced the risk of type II statistical errors. We also chose these cutoffs to be consistent with a similar study from our lab which has been published previously (Von Ruff et al., 2025). Over‐representation analysis (ORA) was performed on DE genes using clusterProfiler and the enrichplot package in R, and referencing both Gene Ontology: Biological Processes and Reactome curated gene sets to identify and characterize functional changes to the transcriptome (Wu et al., 2021; Yu et al., 2012). To identify coordinated pathway‐level changes to the transcriptome we also utilized Gene Set Enrichment Analysis (GSEA). Transcripts were pre‐ranked according to log_2_FC and GSEA was performed using Broad Institute's GSEA software (v4.3.2) and the Reactome and Gene Ontology: Biological Processes gene set databases (Subramanian et al., 2005). Significantly enriched gene sets were identified as those with an FDR < 0.1.

### 2.8 Statistics

For the disuse phase, changes in continuous variables, including unilateral knee extension 1RM, unilateral isometric force production, and leg lean mass for both legs, were assessed between Day 0 and Day 7. Paired *t*‐tests were used to determine statistical significance, with a threshold of *p* < 0.05. Gene expression changes in the disuse leg (left leg) from Day 0 to Day 7 were analyzed. Transcripts were considered differentially expressed if their absolute log_2_FC exceeded 0.4 and the false discovery rate (FDR) was less than 0.1.

During the rehabilitation phase, continuous variables, including unilateral knee extension 1RM, unilateral isometric force production, and leg lean mass for both legs, were assessed at two timepoints: Day 7A versus Day 7B and Day 7A versus Day 21. Paired *t*‐tests were conducted, with statistical significance defined as *p* < 0.05. Gene expression changes in the disuse leg (left leg) were evaluated between Day 7A and Day 7B separately for the resistance exercise group and the walking control group. Transcripts were considered differentially expressed if their absolute log_2_FC exceeded 0.4 and the FDR was less than 0.1. All statistical analyses were performed in either the R programming language or GraphPad Prism 10.

## 3 RESULTS

### 3.1 Seven days of leg suspension lead to reductions in knee extension strength and isometric force production

All study participants completed 7 days of disuse via ULLS where muscle strength, size, and mRNA expression were quantitatively assessed in both the disuse and control leg. Following 7 days of ULLS, participants exhibited a 14.4% decrease in knee extension strength (45.0 ± 15.8 kg vs. 38.5 ± 17.7 kg, *p* < 0.05) and a 13.3% decrease in 60° isometric force production (198.4 ± 56.9 N.m. vs. 172 ± 51.3 N.m., *p* < 0.05) in the disused leg (Table 2). In the control leg, no significant changes occurred in either knee extension strength or isometric force production. Additionally, no significant changes were detected in lean mass measured via dual x‐ray absorptiometry (DEXA) after 7 days of ULLS in either the disuse or control leg. The significant reduction in knee extension strength and isometric force in the disused leg, without a simultaneous decline in the control leg, demonstrates the effectiveness of our disuse model in sustaining non‐weight‐bearing conditions for the disused leg while permitting regular activity in the control leg. Additionally, our disuse protocol resulted in a 10% decrease in Type 2A and an 8% decrease in Type 2X muscle fiber cross‐sectional area in our participants, which was previously reported in a separate publication (Kilroe et al., 2025).

**TABLE 2:** Pre‐post disuse changes in leg lean mass, knee extension strength, and knee isometric force.

Subject characteristics	Pre‐disuse	Post‐disuse
Leg lean mass (DIS), g	7873 ± 1859	7890 ± 1900
Leg lean mass (CON), g	7928 ± 1849	7897 ± 1822
Knee extension 1RM (DIS), kg*	45.0 ± 15.8	38.5 ± 17.7
Knee extension 1RM (CON), kg	47.6 ± 13.6	47.1 ± 15.6
Knee isometric force (DIS), N.m.*	198.4 ± 56.9	172.0 ± 51.3
Knee isometric force (CON), N.m.	186.6 ± 46.6	181.2 ± 46.9

*Note*: Values are means ± SD.

Abbreviations: 1 RM, one‐repetition maximum; CON, control leg; DIS, disuse leg.

* *p* < 0.05.

### 3.2 Disuse alters the expression of mRNA's associated with mitochondrial function, inflammation, and apoptosis

**FIGURE 2:**
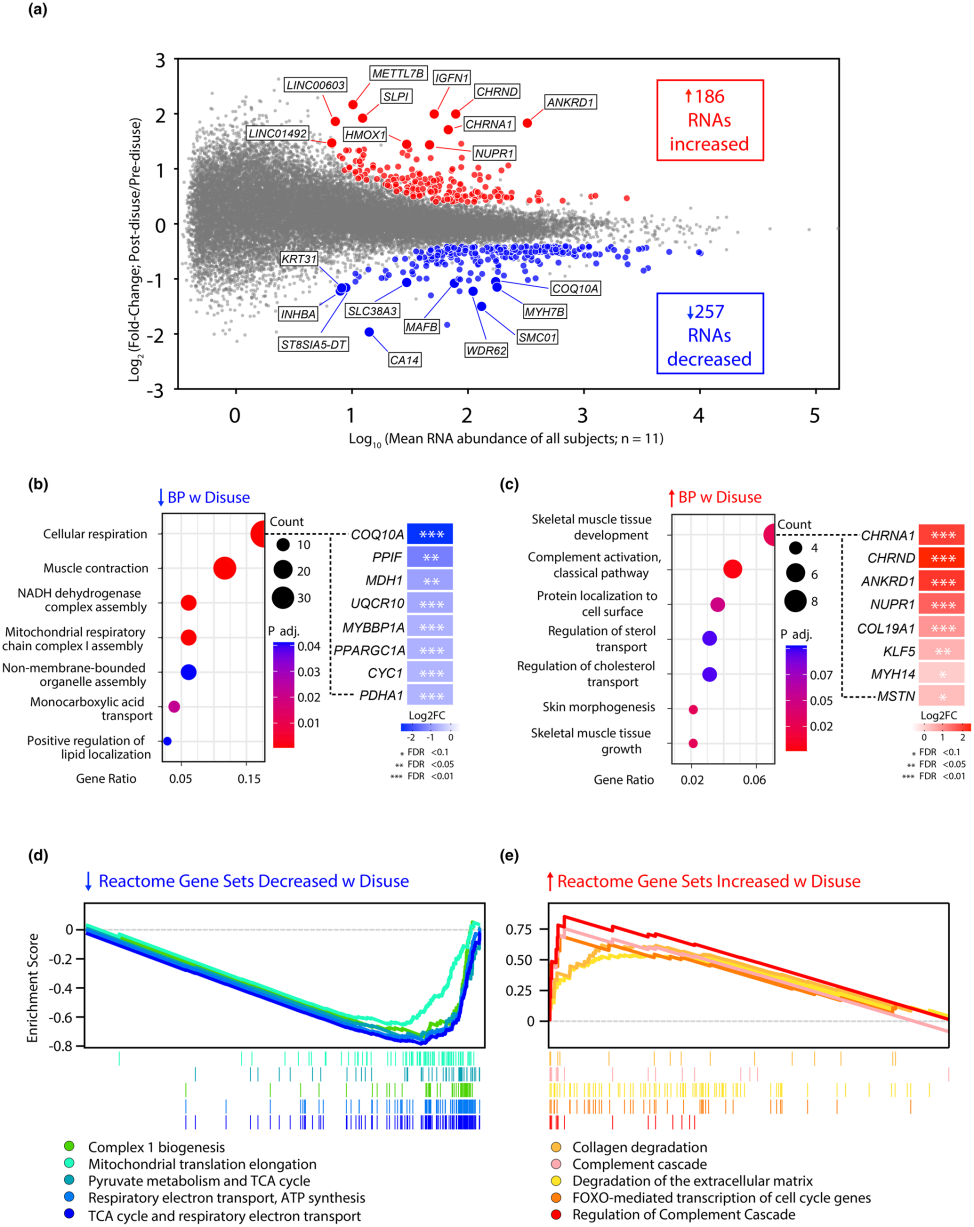
Changes in muscle mRNA expression post‐disuse. We performed global RNA‐seq analysis on human vastus lateralis muscle following seven unilateral leg disuse. (a) MA plot (log_2_ fold change (*M* values, *y*‐axis) versus log‐intensity averages (*A* values, *x*‐axis)) of differences in RNA abundance on Day 0 versus Day 7A of ULLS. Gray circles represent RNA transcripts that did not meet the criteria for differential expression (FDR < 0.1; |L2FC| > 0.4), red symbols represent RNAs that significantly increased with disuse, and blue circles represent RNAs that significantly decreased with disuse. The larger circles and labels note the top 10 upregulated (red) and downregulated (blue) transcripts with disuse. (b and c) Simplified dot plots of overrepresented GO:BP terms determined from significantly downregulated (b) and upregulated (c) genes following disuse and heatmaps of the most enriched transcripts in those pathways *FDR < 0.1, **FDR < 0.05, ***FDR < 0.01. (d and e) GSEA plots of top Reactome Gene Sets decreased (d) and increased (e) following disuse. BP, biological processes; GSEA, gene set enrichment analysis.

To gain insight into the disuse‐induced transcriptomic changes associated with the decreases in muscle strength, we performed global RNA sequencing on skeletal muscle biopsies of the vastus lateralis at Day 0 and Day 7A of disuse. Seven days of disuse had a robust effect on the skeletal muscle transcriptome, with a total of 443 RNAs being differentially expressed (log_2_FC |0.4|, FDR < 0.1) (Figure 2a, Table S1). Of the 443 RNAs differentially expressed, 257 of them were downregulated and 186 were upregulated. Over‐representation analysis (ORA) was applied to the differentially expressed genes to identify pathways that were enriched following disuse.

ORA revealed a robust downregulation of mRNAs related to mitochondrial function including cellular respiration (GO:0045333, FDR < 0.01) and NADH dehydrogenase complex assembly (GO:0010257, FDR < 0.01) among the downregulated genes. (Figure 2b, Table S2). The repression of these biological processes was particularly driven by reduced abundance of mRNAs transcribed from various aspects of mitochondrial function including oxidative phosphorylation (*COX10*), TCA cycle (*MDH1*), and electron transport chain components (*COQ10A* and *UQCR10*) (Buettner et al., 2016; Lian et al., 2022; Xirouchaki et al., 2021). Notably, peroxisome proliferator‐activated receptor‐γ coactivator‐1α (*PPARGC1A*) abundance was significantly reduced following disuse (−0.55 log_2_FC, FDR < 0.001). *PPARGC1A* is considered the master regulator of mitochondrial biogenesis and has important functions in reducing reactive oxygen species production, inflammation, and plays a crucial role in the adaptative response to aerobic exercise (Lira et al., 2010). Additionally, biological process “muscle contraction” (GO:0006936, FDR < 0.01) was significantly over‐represented among the downregulated genes following disuse. Specifically, reduced abundance of transcripts involved in contractile proteins/sarcomere structure (*MYL2*, *MYL3*, *MYL6B*, *MYH7*, *TCAP*, and *MYOT*), energy metabolism (*PGAM2* and *CKMT2*), and neuromuscular junction (*GRIP2* and *KCND3*) was observed following disuse. These data indicate that reduced expression of transcriptomic markers of mitochondrial function and muscle contraction may play a role in loss of strength and force production during inactivity.

ORA of the upregulated genes revealed that biological processes related to skeletal muscle tissue development, complement cascade, and inflammation were over‐represented (Figure 2c, Table S2). The most over‐represented biological processes included “Skeletal muscle tissue development” (GO:0007519, FDR < 0.05), “Complement activation classical pathway” (GO:0006958, FDR < 0.01), “Protein localization to cell surface” (GO:0034394, FDR < 0.1), and “Regulation of sterol transport” (GO:0032371, FDR < 0.1). The increase in the skeletal muscle tissue development pathway is partly driven by the increased expression of transcripts associated with neuromuscular junction components (*CHRNA1* and *CHRND*), extracellular matrix (COL19A1), and mechanotransduction (ANKRD1). Upregulation of this pathway potentially suggests a compensatory mechanism aimed at maintaining neuromuscular junction components and extracellular matrix and function under unloaded conditions to facilitate adaptation for subsequent reloading. Furthermore, increases in immune signaling and inflammation were partly driven by increased expression of mRNAs associated with complement components (*CR1*, *C3*, *C1QC*, *C1QB*, *C1R*, and *C1S*), proinflammatory cytokines (*IL18*), and nuclear factor kappa‐light‐chain‐enhancer of activated B cells (NF‐kB) pathway components. The activation of the NF‐kB pathway has been observed in several models of muscle atrophy including hindleg unloading and cast immobilization (Jackman et al., 2013). These results suggest that skeletal muscle disuse increases transcriptional activity related to immune signaling and inflammation.

### 3.3 Skeletal muscle disuse leads to the downregulation of gene sets involved in mitochondrial function and upregulates gene sets involved in immune signaling and cell death

To provide additional insight into the molecular mechanism behind skeletal muscle disuse at the mRNA level, Gene Set Enrichment Analysis (GSEA) was used to determine which gene sets are positively and negatively enriched following 7 days ULLS. In agreement with the ORA results, GSEA revealed that gene sets related to mitochondrial function were among the most negatively enriched following disuse (Figure 2d, Table S3). Most negatively enriched gene sets included “complex 1 biogenesis” (NES = −2.81, FDR < 0.0001), “mitochondrial translation” (NES = −2.72, FDR < 0.001), “Pyruvate metabolism and TCA cycle” (NES = −2.81, FDR < 0.001), “respiratory electron transport ATP synthesis” (NES = −3.12, FDR < 0.001), and “TCA cycle and respiratory electron transport” (NES = −3.36, FDR < 0.001). Mitochondrial dysfunction is considered a hallmark of skeletal muscle disuse and is characterized by reductions in mitochondrial protein synthesis rates, increased mitochondrial ROS production, and disrupted mitochondrial fusion‐fission dynamics (Hyatt et al., 2019; Ji & Yeo, 2019). Conversely, GSEA revealed that gene sets related to collagen formation/degradation, ECM degradation, immune signaling, and apoptosis were upregulated following disuse (Figure 2e, Table S3). Among the most positively enriched gene sets included “collagen degradation” (NES = 2.16, FDR < 0.001), “complement cascade” (NES = 1.89, FDR < 0.05), “degradation of the extracellular matrix” (NES = 1.81, FDR < 0.05), “FOXO‐mediated transcription of cell cycle genes” (NES = 1.79, FDR < 0.1), and “regulation of complement cascade” (NES = 2.16, FDR < 0.001). These data further indicate that skeletal muscle disuse reduces gene expression related to mitochondrial function and leads to increases pathways involved in immune signaling and cell death.

### 3.4 A single bout of resistance exercise induces robust changes to the transcriptome in both the disused and control leg

**FIGURE 3:**
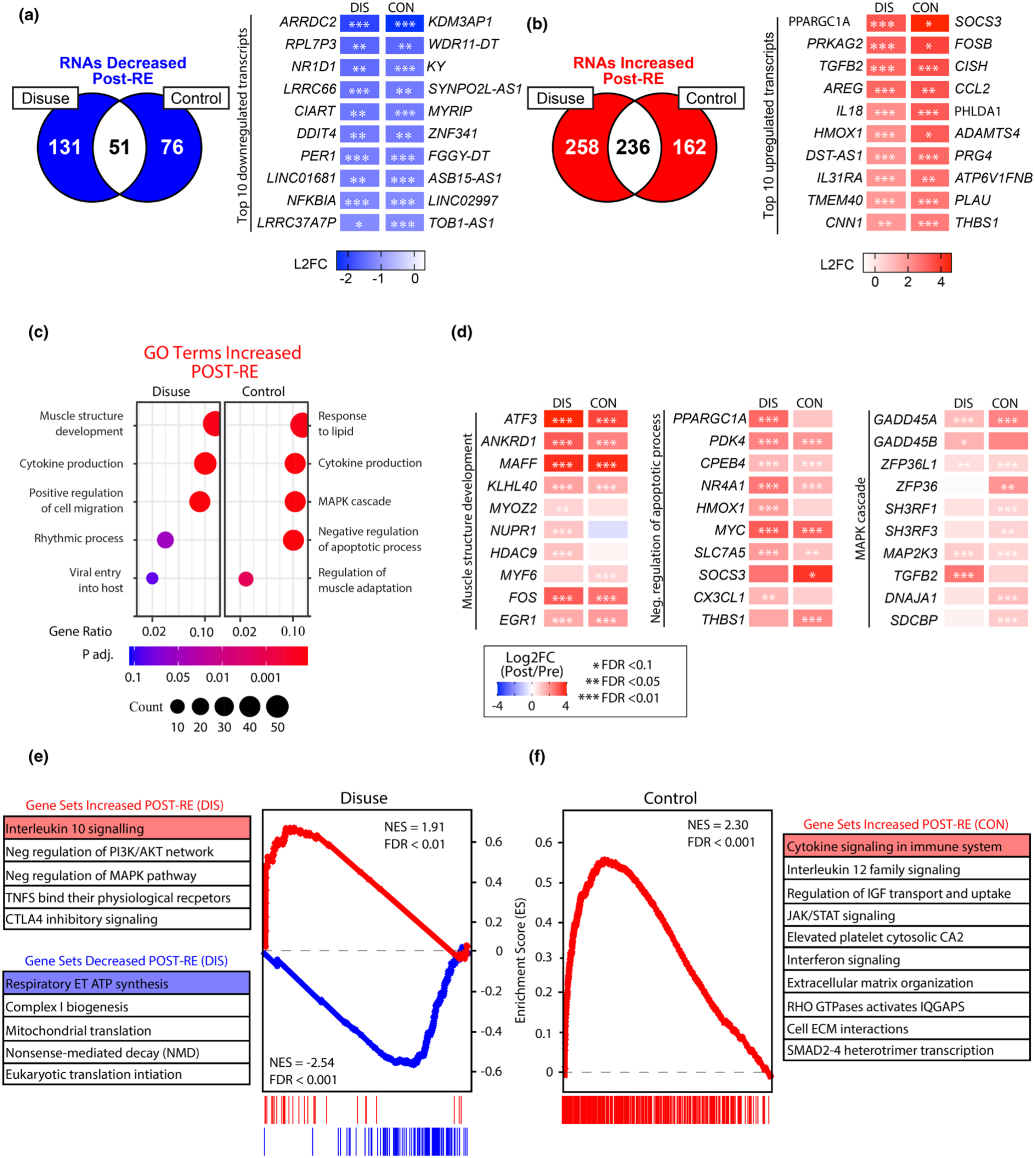
Unique transcriptome alterations following a single bout of resistance exercise in disused and control skeletal muscle (Day 7A vs. Day 7B). (a and b) Venn diagrams and heatmaps of DE genes that decreased (a) or increased (b) following a single bout of resistance exercise (FDR < 0.1; |FDR| > 0.4). (c) Simplified dot plots of overrepresented GO:BP terms determined from significantly increased genes following a single bout of resistance exercise. (d) Heat maps of select differentially expressed genes following RE in both disuse and control legs. (e and f) Gene Set Enrichment Analysis (GSEA) plots of top Reactome gene sets increased and decreased following a single bout of resistance exercise grouped by disused or control leg.

Immediately following the disuse phase, participants randomized to the RE group completed their first rehabilitation session, and bilateral skeletal muscle biopsies of the vastus lateralis were obtained 2 h postexercise (Day 7B). Global RNA sequencing was performed on the skeletal muscle biopsies postexercise to determine the effect of a single bout of resistance exercise on the transcriptome. In the disuse leg, a single bout of resistance exercise led to the differential expression of 675 RNAs, with 494 upregulated and 181 downregulated. In the control leg, a single bout of resistance exercise resulted in the differential expression of 525 RNAs, with 398 upregulated and 127 downregulated. To better understand how resistance exercise is uniquely altering the transcriptome in both the disuse and control leg, we separated the differentially expressed genes that were only expressed in either the disuse or control leg. This analysis revealed that the disuse leg experienced a greater transcriptional response following a single bout of resistance exercise, with 131 versus 76 RNAs uniquely downregulated and 258 versus 162 uniquely upregulated (Figure 3a,b, Table S1). In the disuse leg, transcripts involved in mTORC1 inhibition and skeletal muscle atrophy, including DNA Damage Inducible Transcript 4 (*DDIT4*, also known as *REDD1*) were among the top 10 downregulated transcripts. Interestingly, a downregulation of transcripts associated with circadian rhythm (*PER1* and *CIART*) and calcium homeostasis (*NR1D1*) is observed in the disuse leg after exercise (Boulinguiez et al., 2022; Britto et al., 2014; Malhan et al., 2023) (Figure 3a). In the control leg, none of the top 10 uniquely downregulated genes have been extensively studied in skeletal muscle. Therefore, we included genes common to both the disuse and control leg to gain a better understanding of the transcriptional response to an acute bout of resistance exercise in the control leg. Interestingly, MSTN (also known as myostatin), a negative regulator of muscle growth, was among the top 10 most downregulated genes in the control leg (Rodriguez et al., 2014). The downregulation of muscle atrophy‐related transcripts in the disuse leg and the decrease in myostatin in the control leg may suggest these genes are mediators of the beneficial effects of acute resistance exercise. Additionally, the observed downregulation of genes related to circadian rhythm and calcium homeostasis in the disuse leg implies lingering effects, possibly reflecting an adaptive response to altered physiological demands following disuse.

The top 10 upregulated genes in the disuse leg in response to a bout of resistance exercise included transcripts involved in growth factors (*TGFB2* and *AREG*), metabolic regulation (*PPARGC1A*, *PRKAG2*, and *HMOX1*), and immune signaling and inflammation (*IL18* and *IL31RA*) (Figure 3b). In contrast, in the control leg, the top 10 upregulated were involved in the regulation of the inflammatory response (*SOCS3*, *FOSB*, and *CCL2*) and extracellular matrix remodeling (*ADAMTS4*, *PRG4*, and *PLAU*). The upregulation of distinct genes in the disuse leg and control leg following acute resistance exercise suggests unique molecular responses influenced by disuse and immediate exercise adaptation, respectively. In the disuse leg, some of the distinctly regulated pathways were related to energy metabolism, immune modulation, and tissue repair, while the control leg exhibited changes in inflammation, transcriptional regulation, and tissue remodeling pathways. These results highlight the complex interplay orchestrating skeletal muscle adaptation during and after exercise.

To investigate which pathways were uniquely altered in the disuse and control leg following a single bout of resistance exercise, we utilized ORA on both GO:BP and Reactome pathways (Figure 3c, Table S2). This analysis revealed that biological processes “muscle structure development” (GO:0061061, FDR < 0.01), “cytokine production” (GO:0001816, FDR < 0.01), “positive regulation of cell migration” (GO:0030335, FDR < 0.01), “rhythmic process” (GO:0048511, FDR < 0.1), and “viral entry into host cell” (GO:0046718, FDR < 0.1) were among the most enriched in the upregulated genes in the disuse leg in response to exercise. In the control leg, biological processes “response to lipid” (GO:0033993, FDR < 0.01), “cytokine production” (GO:0001816, FDR < 0.01), “MAPK cascade” (GO:0000165, FDR < 0.01), “negative regulation of apoptotic process” (GO:0043066, FDR < 0.01), and “regulation of muscle adaptation” (GO:0043502, FDR < 0.01). Common themes among both legs were the enrichment in biological processes involved in muscle structure development, negative regulation of cell death, and MAPK signaling (Figure 3d). The upregulation of the muscle structure development pathway was partially driven by transcripts involved in early stress response (*ATF3*, *MAFF*, *FOS*, and *EGR1*) and sarcomere organization (*ANKRD1* and *KLHL40*) regardless of prior disuse or normal activity (Rundqvist et al., 2019; Sabaratnam et al., 2019; Solagna et al., 2020). However, transcripts involved in stress response (*MYOZ2*), cellular homeostasis (*HDAC9*), and apoptosis regulation (*NUPR1*) were upregulated only in the disuse leg which may indicate an enhanced stress response to counteract the effects of disuse.

To further investigate the effects of an acute bout of resistance exercise on the skeletal muscle transcriptome, we performed gene set enrichment analysis (GSEA) of global alterations in RNA abundance. Much like the impact observed on individual RNA transcript abundance (Figure 3a,b, Table S1), this analysis revealed a greater number of differentially enriched gene sets in the disuse leg compared to the control leg (Figure 3e,f, Table S3). Like ORA, positively enriched gene sets in both the disuse and control legs were involved in interleukin signaling and cytokine production. However, in contrast to ORA, GSEA revealed that gene sets involved in the negative regulation of two crucial intercellular signaling cascades involved in regulating muscle growth, MAPK and PI3K/AKT, were among the top positively enriched gene sets in the disuse leg. In contrast, the control leg exhibits unique upregulation of gene sets related to insulin‐like growth factor transport, JAK/STAT signaling, interferon signaling, extracellular matrix organization, and SMAD2‐4 heterodimer transcription, suggesting a more robust transcriptional response to an acute bout of resistance exercise in the control leg compared to the disuse leg. In parallel to our positively enriched gene sets, GSEA revealed that gene sets related to mitochondrial function and muscle protein synthesis were negatively enriched in the disuse leg. There were no significant negatively enriched gene sets following an acute bout of resistance exercise in the control leg. These data support what was observed in our differential gene expression analysis of lingering effects following disuse, leading to a less robust transcriptional response to an acute bout of resistance exercise than the control leg.

### 3.5 A single bout of moderate‐paced walking has minimal effect on the skeletal muscle transcriptome

**FIGURE 4:**
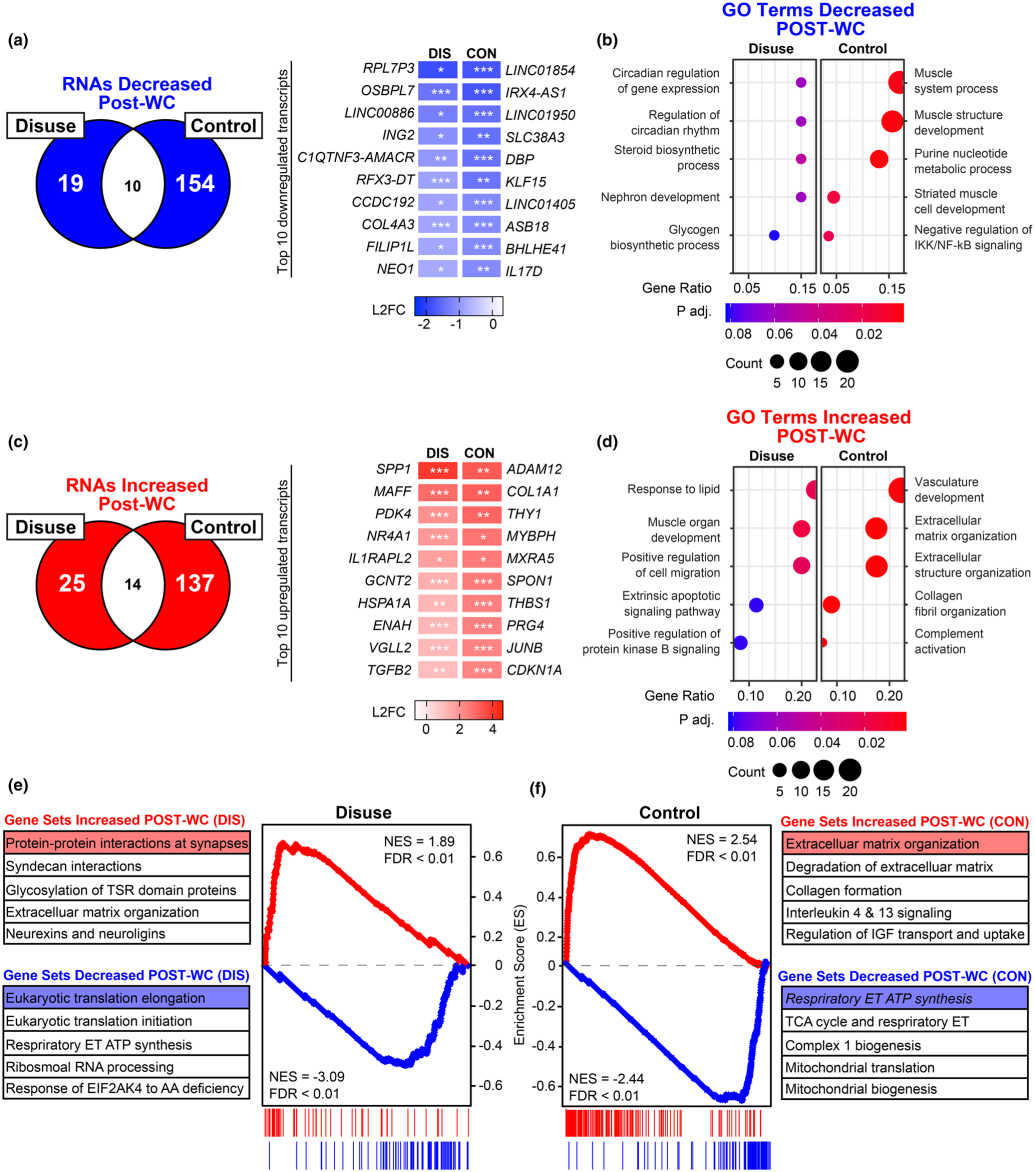
Unique transcriptome alterations following a single bout of treadmill walking in disused and control skeletal muscle (Day 7A vs. Day 7B). (a and c) Venn diagrams and heatmaps of DE genes that decreased (a) or increased (c) following a single bout of treadmill walking (FDR < 0.1; |FDR| > 0.4). (b and d) Simplified dot plots of overrepresented GO:BP terms determined from significantly different transcripts that decreased (b) or increased (d) following a single bout of treadmill walking. (e and f) Gene Set Enrichment Analysis (GSEA) plots of top Reactome gene sets increased and decreased following a single bout of resistance exercise grouped by disused or control leg.

Promptly following disuse, participants in the WC group completed a 30‐min moderately paced treadmill walk. Following the rehabilitation session, bilateral skeletal muscle biopsies of the thigh were obtained 2 h postexercise (Day 7B). Global RNA sequencing revealed a smaller transcriptional response to exercise when compared to the RE group. Additionally, in contrast to the RE group, walking had a minimal effect on the transcriptome in the disuse leg, with 39 significantly upregulated transcripts and 19 significantly downregulated. However, walking still had a modest effect on the transcriptome in the control leg, with 151 significantly upregulated transcripts and 164 significantly downregulated (Table S1). To identify the unique transcriptional response to walking in the disuse and control leg, we examined which DE transcripts were exclusively expressed in either the disuse or control leg (Figure 4a,c). In the disused leg, none of the top 10 most downregulated genes have been studied in skeletal muscle, and their function within skeletal muscle remains elusive. For the control leg, transcripts involved in glutamine transport (*SLC83A*), substrate metabolism (*KLF15*), and protein ubiquitination (*ASB18*) were among the top 10 uniquely downregulated transcripts following an acute bout of treadmill walking (Hirata et al., 2019; Ma et al., 2021; Rubio‐Aliaga & Wagner, 2016). In addition to the downregulated transcripts, an acute bout of treadmill walking uniquely upregulated transcripts associated with metabolic regulation (*PDK4*), stress response/homeostasis (*MAFF* and *HSPA1A*) and myogenesis (*NR4A1*) in the disused leg (Pan et al., 2019; Senf, 2013; Thoudam et al., 2019). In the control leg, transcripts related to extracellular matrix remodeling (*ADAM12*, *COL1A1*, and *THY1*), muscle structure (*MYBPH*, *MXRA5*, and *SPON1*), and stress response (*CDKN1A*) were uniquely upregulated following an acute bout of treadmill walking.

We then performed ORA of the differentially expressed transcripts to determine if any pathways were significantly altered following treadmill walking (Figure 4b,d, Table S2). This analysis revealed that biological processes “circadian regulation of gene expression” (GO:0032922, FDR < 0.1), “regulation of circadian rhythm” (GO:0001816, FDR < 0.1), “steroid biosynthetic process” (GO:0006694, FDR < 0.1), “nephron development” (GO:0072006, FDR < 0.1), and “glycogen biosynthetic process” (GO:0005978, FDR < 0.1) were among the most enriched in the upregulated pathways in the disuse leg. In the control leg, biological processes “muscle system process” (GO:0003012, FDR < 0.01), “muscle structure development” (GO:0061061, FDR < 0.01), “purine nucleotide metabolic process” (GO:0006163, FDR < 0.01), “striated muscle cell development” (GO:0055002, FDR < 0.05), and “negative regulation of IKK/NF‐kB signaling” (GO:0043124, FDR < 0.05). In contrast to the upregulated biological process, ORA revealed that biological processes “response to lipid” (GO:0033993, FDR < 0.05), “muscle organ development” (GO:0007517, FDR < 0.05), “positive regulation of cell migration” (GO:0030335, FDR < 0.05), “extrinsic apoptotic signaling pathway” (GO:0097191, FDR < 0.1), and “positive regulation of protein kinase B signaling” (GO:0051897, FDR < 0.1) were among the most enriched in the upregulated transcripts in the disuse leg following an acute bout of treadmill walking. Alternatively, the most positively enriched biological processes in the control leg included “vasculature development” (GO:0001944, FDR < 0.01), “extracellular matrix organization” (GO:0030198, FDR < 0.01), “extracellular structure organization” (GO:0043062, FDR < 0.01), “collagen fibril organization” (GO:0030199, FDR < 0.01), and “complement activation” (GO:0006956, FDR < 0.01).

To further investigate coordinated changes in biological pathways following an acute bout of treadmill walking, we performed GSEA on the RNA sequencing data in the disuse and control leg of the WC group. This analysis revealed that gene sets involved in extracellular matrix remodeling were positively enriched following an acute bout of treadmill walking (Figure 4e,f, Table S3). However, several gene sets involved in neuromuscular junction dynamics were uniquely upregulated in the disuse leg (Figure 4e, Table S3). In contrast, gene sets involved in interleukin signaling and insulin‐like growth factor transport were uniquely upregulated in the control leg (Figure 4f). Concomitant to our upregulated gene sets, GSEA identified several gene sets involved in mitochondrial function to be negatively enriched in both disuse and control legs. Furthermore, this analysis revealed that gene sets associated with two key steps in muscle protein synthesis, eukaryotic elongation and initiation, were uniquely downregulated in the disuse leg after walking (Vilchinskaya et al., 2023). Collectively, these data suggest that despite the blunted transcriptional response to an acute bout of treadmill walking in the disuse leg, some pathways related to skeletal muscle are still altered.

### 3.6 Molecular time course of rehabilitation

**FIGURE 5:**
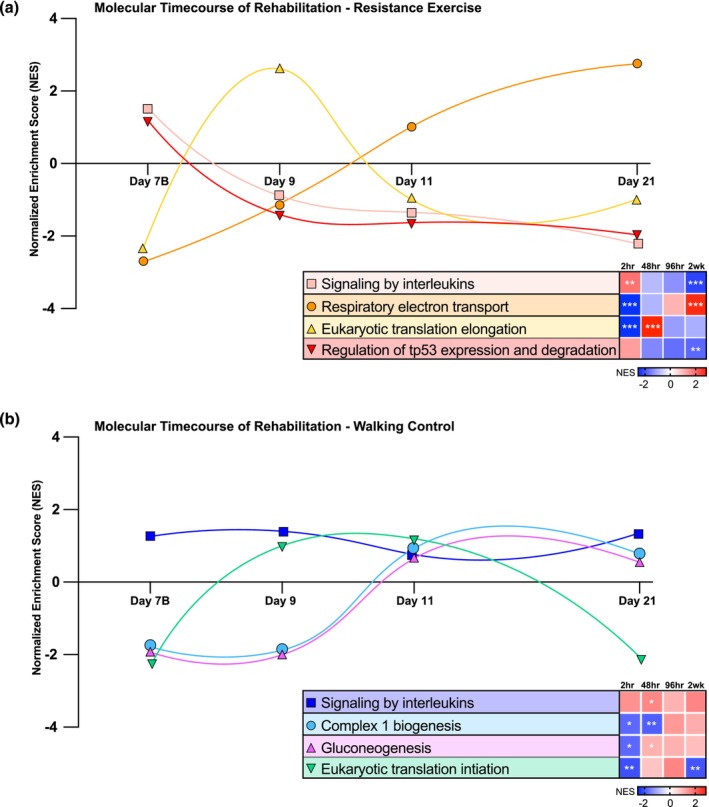
Molecular time course of rehabilitation. Significantly enriched Reactome gene sets from skeletal muscle biopsies 2 h post‐resistance exercise (a) and treadmill walking (b) on Day 7B, Day 9, Day 11, and Day 21. *FDR < 0.1, **FDR < 0.05, ***FDR < 0.001.

We sought to identify biological pathways involved in the early phase recovery of skeletal muscle following disuse and observe how they are altered over the course of a 2‐week rehabilitation intervention. To do this, we performed GSEA on global RNA‐seq data at 2 h (1 rehabilitation session), 48 h (2 rehabilitation sessions), 96 h (3 rehabilitation sessions), and 2 weeks (7 rehabilitation sessions) post rehabilitation in both the RE and WC groups (Figure 5) on muscle biopsies from the left leg. Following the analysis, we identified gene sets involved in mitochondrial function, protein synthesis/breakdown, interleukin signaling, and glucose metabolism were common themes at most timepoints. Therefore, for the RE group we selected Reactome gene sets “signaling by interleukins”, “respiratory electron transport”, “eukaryotic translation elongation”, and “regulation of tp53 expression and degradation” to observe over the course of the 2‐week resistance exercise rehabilitation intervention (Figure 5a). This analysis revealed that gene set “signaling by interleukins” was significantly upregulated after an acute bout of resistance exercise (NES = 1.85, FDR < 0.05), trended downward at 48 h and 96 h, and was significantly downregulated at 2 weeks (NES = −2.23, FDR < 0.0001) post resistance exercise intervention. In contrast, gene set “respiratory electron transport” was significantly downregulated 2 h (NES = 2.37, FDR < 0.0001) post resistance exercise, trended upward at 48 h and 96 h, and was significantly upregulated at 2 weeks (NES = 2.78, FDR < 0.0001) post resistance exercise intervention. Gene set “eukaryotic translation elongation” was significantly downregulated 2 h (NES = −2.51, FDR < 0.0001) post resistance exercise followed by a rapid upregulation at 48 h (NES = 2.84, FDR < 0.0001). Further, gene set “regulation of tp53 expression and degradation” trended upward 2 h post RE, followed by a steady downregulation at 48 h and 96 h, and was significantly downregulated at 2 weeks post RE (NES = −1.82, FDR < 0.05). Lastly, on average, knee extensor 1RM increased in the RE group in the disuse leg. All post‐rehabilitation muscle phenotype outcomes are presented in Table 3.

**TABLE 3:** Post‐disuse to post‐rehabilitation changes in leg lean mass, knee extension strength, and knee isometric force stratified by leg and rehabilitation protocol.

Subject characteristics	Post‐disuse (RE)	Post‐rehab (RE)	Post‐disuse (WC)	Post‐rehab (WC)
Leg lean mass (DIS), g	7781.2 ± 2278.6	7562.2 ± 2173.6	8020.8 ± 1579.2	8065.8 ± 1464.9
Leg lean mass (CON), g	7924.5 ± 2259.5	7703.4 ± 2136.9	7863.6 ± 1383.2	7830.8 ± 1292.2
Knee extension 1RM (DIS), kg	35.3 ± 20.2	39.9 ± 14.0*	42.4 ± 15.6	45.4 ± 17.4
Knee extension 1RM (CON), kg	48.7 ± 17.5	46.5 ± 15.3	45.2 ± 14.9	47.0 ± 14.1
Knee isometric force (DIS), N.m.	175.3 ± 67.3	175.6 ± 28.2	168.6 ± 39.7	187.3 ± 50.9
Knee isometric force (CON), N.m.	193.7 ± 61.7	192.7 ± 36.5	168.8 ± 30.2	190.9 ± 34.3

*Note*: Values are means ± SD.

Abbreviations: 1 RM, one‐repetition maximum; CON, control leg; DIS, disuse leg; RE, resistance exercise group, WC, walking control group.

* *p* < 0.05.

For the WC group, we selected Reactome gene sets “signaling by interleukins”, “complex 1 biogenesis”, “gluconeogenesis”, and “eukaryotic translation initiation” to follow over the course of the 2‐week treadmill walking intervention (Figure 5b). In contrast to the RE group, gene set “signaling by interleukins” was significantly upregulated 48 h (NES = 1.56, FDR < 0.1) post walking intervention and trended upward at all other timepoints. Gene set “complex 1 biogenesis” was significantly downregulated at 2 h (NES = −1.79, FDR < 0.1) and 48 h (NES = −1.91, FDR < 0.05) post treadmill walking. Additionally, gene set “complex 1 biogenesis” trended upward at 96 h and 2 weeks post treadmill walking but failed to reach statistical significance. Gene set “gluconeogenesis” was significantly downregulated at 2 h (NES = −1.80, FDR < 0.1) post and 48 h (NES = −1.75, FDR < 0.1) rehabilitation. Finally, gene set “eukaryotic translation initiation” was significantly downregulated at 2 h (NES = −2.04, FDR < 0.05) post treadmill walking, trended upward at 48 h and 96 h, and was significantly downregulated at 2 weeks (NES = −2.01, FDR < 0.05).

## 4 DISCUSSION

In the present study, our goal was fourfold: (1) characterize the effects of 7 days of disuse via ULLS on the transcriptome in healthy middle‐aged adults, (2) examine and compare how an acute bout of resistance exercise and treadmill walking uniquely alter the transcriptome immediately following disuse, (3) compare how the transcriptome of disused and healthy skeletal muscle respond to rehabilitation, and (4) map out the transcriptomic time course of rehabilitation in skeletal muscle following disuse. To do this, we utilized global RNA‐Seq data from human skeletal muscle biopsies from both the disuse and control leg at crucial timepoints during disuse and rehabilitation. Our findings revealed widespread changes to the transcriptome following 7 days of ULLS. Notably, 7 days of disuse led to the negative enrichment of several gene sets associated with mitochondrial function. In contrast, 7 days of disuse led to the positive enrichment of gene sets involved in immune signaling, inflammation, and apoptosis. Additionally, an acute bout of resistance exercise had a more profound effect on the transcriptome than treadmill walking. Further investigation on how the transcriptome of the disuse and control leg responds to an acute bout of resistance exercise revealed lingering effects of disuse, including the downregulation of transcripts and pathways involved in mitochondrial function and muscle protein synthesis in the disuse leg. Lastly, gene sets involved in mitochondrial function, interleukin signaling, protein synthesis/degradation, and glucose metabolism are time sensitive and enriched at different time points of rehabilitation.

Skeletal muscle disuse precipitates numerous deleterious effects that profoundly impact musculoskeletal health and functional well‐being. While our knowledge of the molecular mechanisms of disuse atrophy has grown drastically, the transcriptomic signatures that underly disuse atrophy remain understudied in humans. Therefore, we utilized ORA and GSEA to identify pathways that are altered following 7 days of disuse. This approach revealed several biological processes and gene sets involved in various aspects of mitochondrial function including cellular respiration, TCA cycle, and mitochondrial biogenesis were significantly downregulated following disuse. Interestingly, these data are supported from a previous publication from the same study in which we observed significant reductions in TCA cycle and glycolytic metabolites, including acetyl‐CoA and citric acid. Additionally, when we performed an integrated analysis of altered metabolites and differentially expressed genes, the top downregulated pathway was the TCA cycle (Kilroe et al., 2025). In normal conditions, mitochondria are critical in maintaining cellular homeostasis and skeletal muscle health. However following disuse, mitochondrial dynamics are disrupted contributing to reductions in mitochondrial biogenesis and increased production of reaction oxygen species, and cell death (Chen et al., 2023; Ji & Yeo, 2019). Our findings are supported by previous work where 10 days of bed rest resulted in reductions in mitochondrial respiration, content, and increased emissions of mitochondrial ROS (Standley et al., 2020). Bilet et al. also demonstrated reduced in vivo mitochondrial respiratory capacity following 9 days of ULLS (Bilet et al., 2020). At the transcript level, previous work in humans has shown a downregulation of biological pathways associated with mitochondrial function following 10 days of disuse, including fatty acid degradation, OXPHOS, and TCA cycle (Standley et al., 2020). Concomitant with the downregulated pathways, RNA‐seq revealed that biological pathways related to immune signaling, inflammation, and protein degradation were upregulated following 7 days of ULLS in the disuse leg in our participants. Previous work has demonstrated that inflammation plays a pivotal role in skeletal muscle atrophy by disrupting muscle protein balance (Tavares‐Neto et al., 1986). In the present study, immune signaling and inflammation was mediated through the activation of the complement system. The complement system is composed of more than 40 proteins which are dispersed throughout various body fluids and tissues and plays a pivotal role in the innate immune response (Dunkelberger & Song, 2010). There are three major pathways by which the complement system can be activated: the classical, lectin, and alternative (Tu & Li, 2023). We found that key transcripts involved in the classical pathway of the complement system were upregulated following disuse including *C3*, *C1QB*, *C1QC*, *C1R*, and *C1S*. The upregulation of these transcripts and biological pathways may indicate a protective response or attempt to restore homeostasis in skeletal muscle under conditions of disuse. Previous work has identified that the activation of the complement cascade, specifically the alternative pathway, is crucial for muscle regeneration following injury (Zhang et al., 2017). In contrast, some evidence suggests that the activation of the complement system is associated with muscle loss during aging and disuse. For example, protein abundance of complement factor H (*CFH*) in plasma is elevated in patients more prone to muscle atrophy during bedrest (Murgia et al., 2023). Additionally, increased secretion of complement component 1q (*C1Q*) leads to muscle fibrosis in senescent mice (Horii et al., 2018). Increased *C1Q* expression can lead to the activation of the Wnt pathway in skeletal muscle, which activates forkhead box O (FoxO) signaling, resulting in muscle degradation (Horii et al., 2018; Okada et al., 2015). This aligns with our data as the Reactome gene set “FOXO‐mediated transcription of cell cycle genes” was among the most positively enriched following disuse. Taken together, these data indicate that disuse alters the skeletal muscle transcriptome via reductions in transcripts and pathways associated with mitochondrial function and increases in pathways associated with immune signaling, inflammation, and protein degradation.

An acute bout of resistance exercise following disuse had a significantly greater effect on the skeletal muscle transcriptome than treadmill walking. We found that a single bout of resistance exercise led to ~163% greater number of differentially expressed transcripts (676 vs. 68) than treadmill walking in the disuse leg. Additionally, we found that biological pathways related to mitochondrial function and muscle protein synthesis were negatively enriched following an acute bout of resistance exercise in the disuse leg. In a recent meta‐analysis, disuse and resistance exercise display divergent transcriptomic signatures for many biological processes including mitochondrial function and translation, indicating that disuse is not simply the reverse of resistance exercise but rather that the two are distinct processes (Deane et al., 2021). In contrast, we found several common mitochondrial pathways between disuse and resistance exercise conditions remained downregulated including complex 1 biogenesis, respiratory electron transport, and mitochondrial translation. This discrepancy is likely due to our participants participating in both disuse and rehabilitation conditions consecutively. Moreover, it is probable that a single bout of resistance exercise is insufficient to fully restore mitochondrial function following disuse. Our lab has previously reported that mitochondrial respiration improves following 12 weeks of resistance exercise training (Porter et al., 2015), indicating that prolonged exposure to resistance exercise is required to see meaningful changes in mitochondrial function. We further observed that several gene sets related to translation initiation were altered following a single bout of resistance exercise in the disuse leg. Translation initiation is the rate limiting step in protein synthesis and its related pathways (i.e., PI3K/AKT/mTOR) are crucial regulators of muscle hypertrophy (Augert et al., 1986; Bodine et al., 2001; Rommel et al., 2001). We have reported previously that upstream regulators (AKT) and downstream effectors (i.e., SK61 and 4E‐BP1) of mTOR signaling reach peak phosphorylation 3‐h post resistance exercise (Drummond et al., 2008). In addition, Edman et al. recently reported that mRNAs encoding for translation initiation peak between 3‐ and 8‐h post resistance exercise (Edman et al., 2024). In contrast, we reported that Reactome gene sets “negative regulation of PI3K/AKT network” and “eukaryotic translation initiation” were significantly upregulated and downregulated, respectively. Resistance exercise led to some transcriptomic changes that were common between control and disuse legs, including the downregulation of *MSTN* in both legs. However, other changes occurred in only one leg, including the downregulation of *DDIT4* in the control‐leg and greater decreases in mitochondrial genes in the disuse leg. These findings highlight the complexity of the molecular mechanisms underlying muscle anabolism, suggesting that disuse may alter some of the acute anabolic processes crucial for muscle adaptation.

The transcriptional events that underly skeletal muscle adaptation to resistance exercise are dynamic, particularly in the early phases of training (Murton et al., 2014). However, previous work investigating the effects of resistance exercise on the transcriptome primarily utilizes a pre‐post design, likely missing the key transcriptional events that occur during the initial exposure to resistance exercise (Gordon et al., 2012; Li et al., 2022; Sarto et al., 2022). Furthermore, there is a paucity of research that investigates the transcriptome changes that underpin recovery following short‐term immobilization. Therefore, we sought to map the transcriptomic time course of recovery following short‐term immobilization. Additionally, we sought to characterize how different rehabilitation protocols (i.e., resistance exercise and treadmill walking) affect the transcriptome. GSEA revealed that pathways related to mitochondrial function, inflammation, and translation are altered dynamically throughout the course of rehabilitation and vary depending on rehabilitation protocol. Pathways related to mitochondrial function were downregulated at 2 h in both groups. Our results align with previous work by Murton et al. who observed a reduced abundance of transcripts associated with mitochondrial oxidative phosphorylation 24 h post resistance exercise and remained below basal levels at 48 h (Murton et al., 2014). Similarly, Damas et al. observed that acute resistance exercise downregulates transcripts associated with oxidative metabolism (Damas et al., 2018). The suppression of the respiratory electron transport pathway following acute resistance exercise could be a compensatory mechanism to counteract the increase in ROS production that has been observed with acute exhaustive exercise (Jin et al., 2015). However, in the WC group, the reduction in the electron transport pathway at 2 h may be attributable to prior disuse, during which moderate treadmill walking may have been insufficient to significantly stimulate mitochondrial pathways. After 2 weeks of rehabilitation, the RE and WC groups showed opposing transcriptional responses with mitochondrial function pathways being upregulated and downregulated, respectively. Previously, it was found that 10 weeks of resistance exercise upregulated oxidative metabolism pathways at rest (Damas et al., 2018). Together, these data demonstrate that mRNAs encoding mitochondrial proteins respond dynamically during rehabilitation and are influenced by the type and duration of the exercise protocol.

Similarly, opposing transcriptional responses were observed with interleukin signaling between the RE and WC groups at 2 h, 48 h, and 2 weeks of rehabilitation. The observed upregulation of interleukin signaling pathways at 2 h post‐resistance exercise suggests an acute inflammatory response to the exercise stimulus, which has been observed in previous studies. For example, mRNA content of several inflammatory mediators (IL‐6, IL‐8, and TNFα) is upregulated 3 h post‐resistance exercise (Buford et al., 2009). However, we observed that subsequent resistance exercise downregulated this pathway, ending with significant downregulation at 2 weeks, suggesting a potential adaptation or resolution of the acute inflammatory response over time. Similarly, it has been reported that serum gene expression of IL‐6 and TNFα and TNFα decrease following 6 weeks of resistance exercise (Macêdo Santiago et al., 2018). In contrast, interleukin signaling remained upregulated throughout the course of rehabilitation, reaching peak enrichment at 48 h. In summary, our findings reveal dynamic changes in interleukin signaling pathways throughout the rehabilitation process and demonstrate that resistance exercise exerts anti‐inflammatory effects with prolonged training. Lastly, we saw that pathways associated with translation were downregulated by 2 h in both groups but significantly upregulated by 48 h in the RE group only. As previously noted, the decline in mRNAs linked to translation may be a consequence of prior disuse; nevertheless, repeated resistance exercise significantly upregulates translation pathways.

A unique aspect of this study was the middle‐aged population that was recruited. Most disuse studies recruit young individuals, or in some cases, old individuals. However, there is little available data in middle‐aged adults. This is an important age group because these individuals are nearing the initial stages of age‐related physical decline, and preserving mobility and strength into later years ideally should begin before serious declines occur. While this population is unique, many of the transcriptomic changes observed here are consistent with other studies showing that disuse reduces transcriptional regulation of metabolism‐related genes and increases inflammation‐related genes, and that the reverse is largely true with exercise training (Bilet et al., 2020; Dirks et al., 2016; Sabaratnam et al., 2019; Standley et al., 2020). However, it would be interesting for future work to make direct comparisons between young, middle‐aged, and older adults to see if/when alterations in exercise responses occur in terms of physiological and transcriptomic changes.

While this study provides valuable insights into the transcriptomic responses to disuse and rehabilitation in skeletal muscle, several limitations should be considered. Firstly, it is important to note that transcriptome changes observed in this study may not directly translate to physiological changes, and further validation through functional assays is warranted. Secondly, we did not stratify by sex for any of our analyses, limiting our ability to discern sex‐specific transcriptome changes associated with disuse and rehabilitation. Future investigations should prioritize sex‐stratified analyses to elucidate potential differences in the molecular responses between males and females. The male subjects had a BMI that is considered obese, and this may have influenced the degree of loading and unloading. Additionally, the timing of biopsies during the rehabilitation phase was taken 2 h postexercise and may not fully capture the basal gene expression changes induced by the rehabilitation protocol. For example, various studies have shown that gene expression not only fluctuates for the first several hours postexercise but fluctuates for up to 4 days after a single exercise bout (Kuang et al., 2022; Kusano et al., 2024; Neubauer et al., 2014). Additionally, it has been shown that peak gene expression correlates with exercise‐induced changes in protein expression, but that peak gene expression does not occur at the same timepoints for different genes (Kusano et al., 2024). Thus, caution should be exercised when interpreting the impact of the rehabilitation intervention on baseline gene expression.

Our study highlights the role physical activity can play in mitigating the detrimental effects of inactivity on muscle function. We observed significant losses in leg strength and force production after just 7 days of disuse, highlighting the speed at which these measures can decrease with unloading. Furthermore, our findings suggest that prolonged or more intense rehabilitation strategies may be warranted to fully counteract the lingering effects of disuse. Importantly, our comparison of resistance exercise to treadmill walking revealed that the resistance group exhibited a larger decrease in interleukin signaling pathways and a larger increase in mitochondrial function pathways. These differences between resistance training and walking may provide insight into why resistance training elicits greater increases in muscle strength during periods of rehabilitation.

## Supporting information


Table S1.

Table S2.

Table S3:
